# Boiling as a Means of Keeping Milk

**Published:** 1888-05

**Authors:** 


### BOILING AS A MEANS OF KEEPING MILK.

The souring of milk is caused by the change of the sugar of milk into lactic acid, under the influeuce of the caseine, or cheesy matter, which the milk contains. Boiling, however, deprives caseine of the power of converting the sugar of milk into lactic acid. Now, if the milk be put into bottles which, after being well corked, are placed in a can with cold water, and gradually raised to the boiling point, and after being allowed to cool, be taken out and placed in a cool place, the milk will keep perfect for more than a half a year. If the milk be exposed to the air after boiling, the caseine gradually recovers its power of converting sugar into lactic acid. In this case the boiling has to be repeated every day or every second day, and milk may thus be kept fresh for two months or more.

